# Prevalence, clinical features and severity of allergic rhinitis in elderly: preliminary results

**DOI:** 10.1186/1939-4551-8-S1-A276

**Published:** 2015-04-08

**Authors:** Jose Laerte Boechat, Daniella Moore, Victor Cortes, Simone Pestana, Amanda Seba, Rossana Rabelo, Jose Rodrigo De Moraes, Fernando Carvalho

**Affiliations:** 1 Universidade Federal Fluminense, Brazil

## Background

In the last years, studies have demonstrated that chronic rhinits is a common disorder in elderly patients, although epidemiologic data related to this population is sparing. Indeed, there is no information regarding the prevalence of rhinits in the elderly in Brazil.

The aim of this study was to determine the prevalence, clinic features and severity of allergic rhinitis (AR) in the elderly and compare them with an age-matched group with non-allergic rhinitis (NAR).

## Methods

Prospective study. Geriatric patients with nasal symptoms assisted at Universidade Federal Fluminense (UFF) Immunology Service in Niterói, Brazil were analyzed upon clinical questionnaire, physical examination and skin prick test (SPT).

Diagnosis of AR was made based on characteristic clinical findings and positive SPT result. For the diagnosis of nonallergic rhinitis, it was required a negative SPT result among those with clinical positive findings.

The two groups of patients – those with AR and NAR – were compared in terms of gender, age, clinical features, severity level and duration. Statistical analysis were performed using SPSS 18,0 program.

## Results

Thirty-two individuals above 60 years of age were included in the analysis. The prevalence of allergic rhinitis was 25%. The age in the sample ranged from 61 to 78 yo in the atopic group and from 60 to 85 yo in the non-atopic patients. In AR group, 7 were female (87,5%); of the patients in the group of NAR, 19 were female (79,2%). The most common symptom in atopic patients was nasal congestion following by rhinorrhea and in the other group, nasal congestion following by sneezing. There was no statistic difference between the prevalence of symptoms in both groups (p>0.05). There was no difference between the groups in terms of severity and duration of the symptoms. The majority of patients with AR and NAR (33% and 37, 5%, respectively) were classified as mild / persistent rhinitis.

## Conclusion

To date, the prevalence of allergic rhinitis in elderly patients in our sample was similar to that encountered in general population. Furthermore, clinical findings and severity of the symptoms were similar in AR and NAR patients.

